# Popliteal artery aneurysm with popliteal artery entrapment syndrome: a case report and literature review

**DOI:** 10.3389/fsurg.2026.1795551

**Published:** 2026-03-30

**Authors:** Yiming Ren, Lianrui Guo

**Affiliations:** Department of Vascular Surgery, Xuanwu Hospital, Capital Medical University, Beijing, China

**Keywords:** autologous vein bypass grafting, popliteal artery aneurysm (PAA), popliteal artery entrapment syndrome (PAES), posterior approach for aneurysm decompression, secondary turbulent flow-induced arterial dilation

## Abstract

**Background:**

Popliteal artery entrapment syndrome (PAES) is a rare anatomical anomaly that may rarely lead to secondary popliteal artery aneurysm (PAA). We present a case in which chronic extrinsic compression culminated in a giant PAA, highlighting the diagnostic workflow and surgical strategy.

**Case presentation:**

A 50-year-old man presented with a 2-year history of exercise-induced pain and numbness in the right lower limb. On examination, popliteal and distal pulses were absent. Duplex ultrasound showed a 4.1 cm saccular popliteal artery aneurysm (PAA) containing a 15 mm mural thrombus; CT angiography revealed moderate-to-severe stenosis of the proximal popliteal artery. Intra-operatively, the popliteal artery was found compressed and occluded by the medial head of the gastrocnemius, confirming popliteal artery entrapment syndrome; after partial myotomy to release the vessel, only the eccentric aneurysmal sac wall was excised while the macroscopically normal-appearing arterial wall was preserved, and the artery was reconstructed with an autologous small-saphenous-vein patch angioplasty.

**Discussion:**

PAES-induced PAA is uncommon; failure to recognise the underlying entrapment risks could lead to incorrect endovascular treatment. Open decompression combined with venous bypass remains the gold standard when the artery is structurally damaged.

**Conclusion:**

Clinicians encountering an isolated PAA in a relatively young patient should actively exclude PAES. Timely surgical decompression and revascularisation can prevent thrombo-embolic complications and limb loss.

## 1 Introduction

Popliteal Artery Entrapment Syndrome (PAES) is a rare anatomical anomaly in which an abnormal muscle or fibrous band in the popliteal fossa compresses the popliteal artery or vein, leading to corresponding pathological changes and clinical manifestations; occasionally, the nerve may also be involved. Popliteal artery aneurysm (PAA) is the most serious—though still uncommon—complication of PAES and can ultimately result in limb-threatening ischemia. We report a middle-aged male patient who presented with numbness and pain in the lower limb; imaging studies and intra-operative findings revealed a popliteal artery aneurysm accompanied by popliteal artery entrapment syndrome.

## 2 Case history

The patient was a 50-year-old male who presented with a 2-year history of numbness and pain in the right lower limb without any obvious precipitating cause. Symptoms were significantly aggravated by standing or physical activity and relieved by rest. On physical examination, the popliteal artery, dorsalis pedis artery, and posterior tibial artery were not palpable on the right side. Percussion over the right popliteal fossa exacerbated the numbness in the lower limb. The patient had no history of trauma or surgery in the popliteal region. Laboratory tests, including complete blood count, liver and kidney function, electrolytes, inflammatory markers, electrocardiogram, and chest x-ray, were all within normal limits at admission.

**Figure 1:**
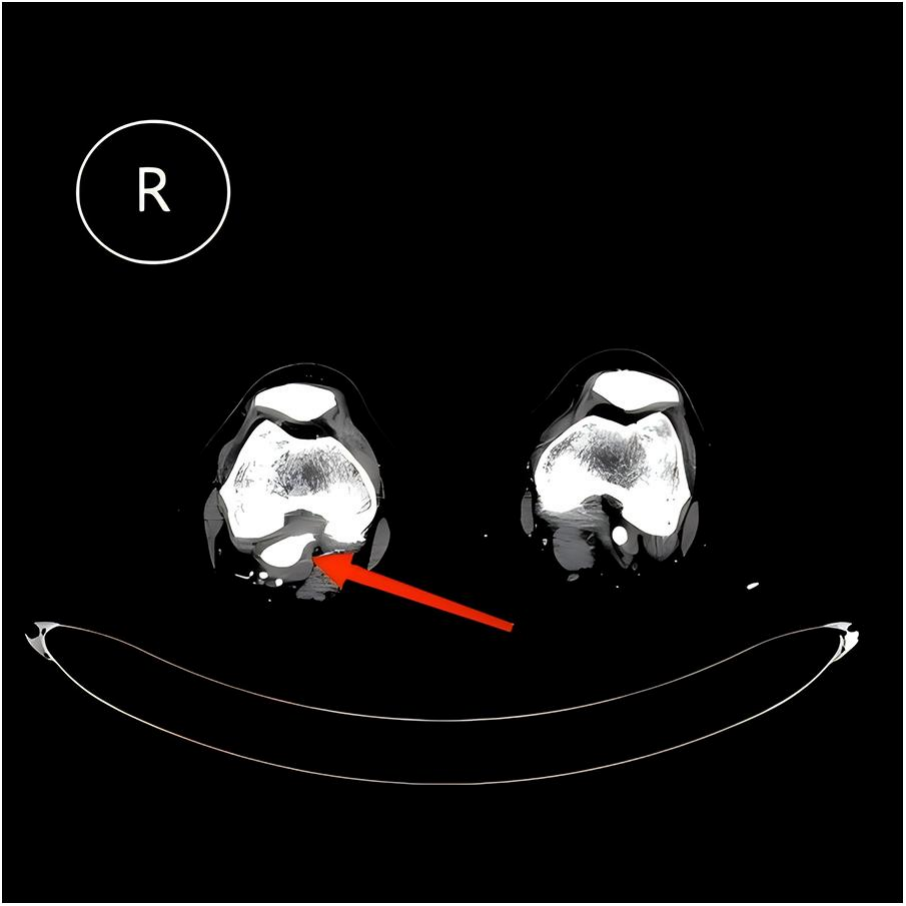
Ultrasound image showing a right popliteal artery aneurysm (4.1 cm) with a 15 mm mural thrombus; the residual lumen measures 2.2 cm. Arrow indicates the popliteal artery aneurysm.

**Figure 2:**
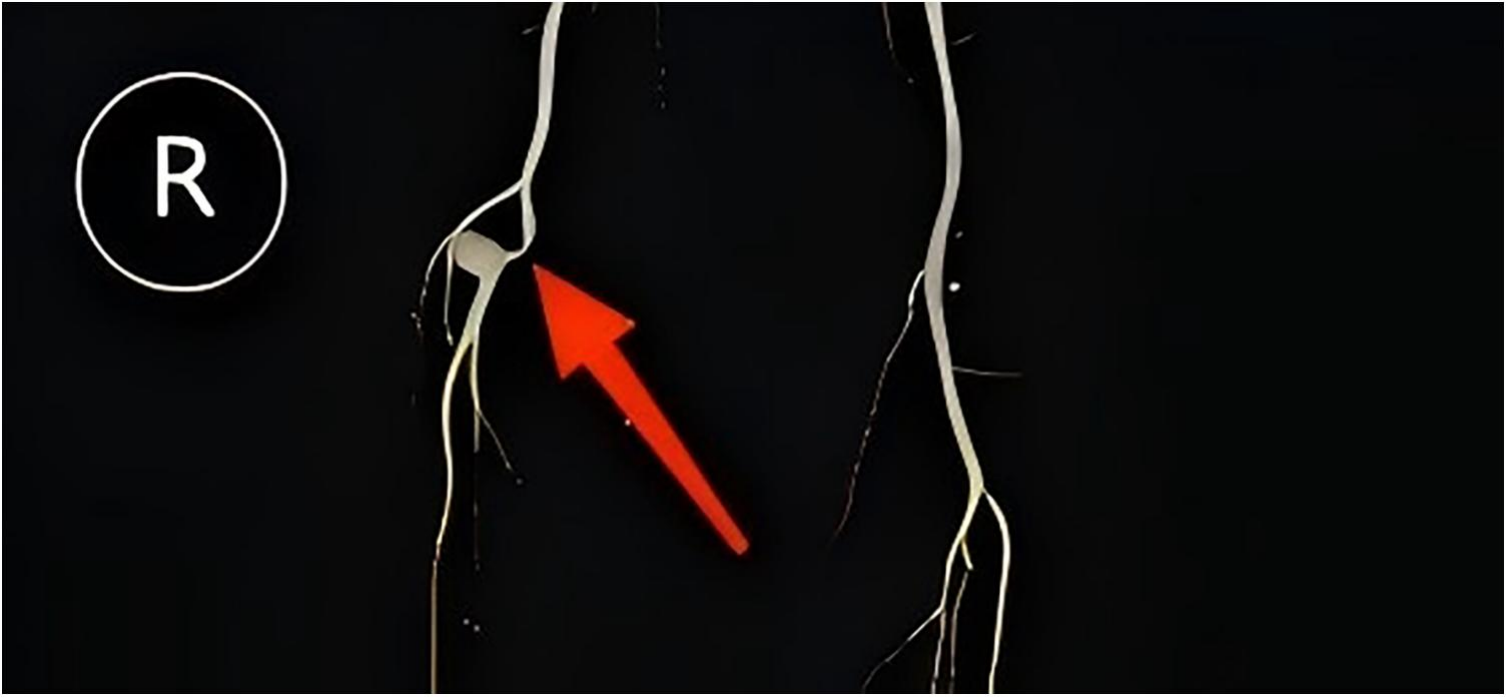
CT angiography showing the aneurysm with a maximum diameter of 3.7 cm and moderate-to-severe extrinsic stenosis of the proximal parent artery. Arrow indicates the compressed and stenotic segment above the popliteal artery aneurysm.

Imaging findings: Preoperative lower extremity ultrasound revealed a focal dilatation of the right popliteal artery, presenting as an aneurysm measuring 4.1 × 3.6 × 3.0 cm, with a 15 mm echogenic mural thrombus attached. The residual lumen measured approximately 2.2 × 1.9 cm, suggesting a right popliteal artery aneurysm with mural thrombus (Figure 1). CT angiography (CTA) of the lower extremity showed a focal outpouching of the right popliteal artery, with a maximum cross-sectional diameter of 3.7 × 3.2 cm. The center of the aneurysm showed contrast enhancement, surrounded by a non-enhancing rim. The proximal segment of the affected artery showed moderate to severe stenosis (Figure 2), likely due to extrinsic compression or arterial wall injury, leading to hemodynamic changes and secondary turbulent flow, resulting in post-stenotic aneurysmal dilation. The patient's primary symptoms were numbness and pain in the right lower limb, which worsened with physical activity. Based on the clinical history and imaging findings, a diagnosis of popliteal artery aneurysm secondary to popliteal artery entrapment syndrome was considered.

**Figure 3:**
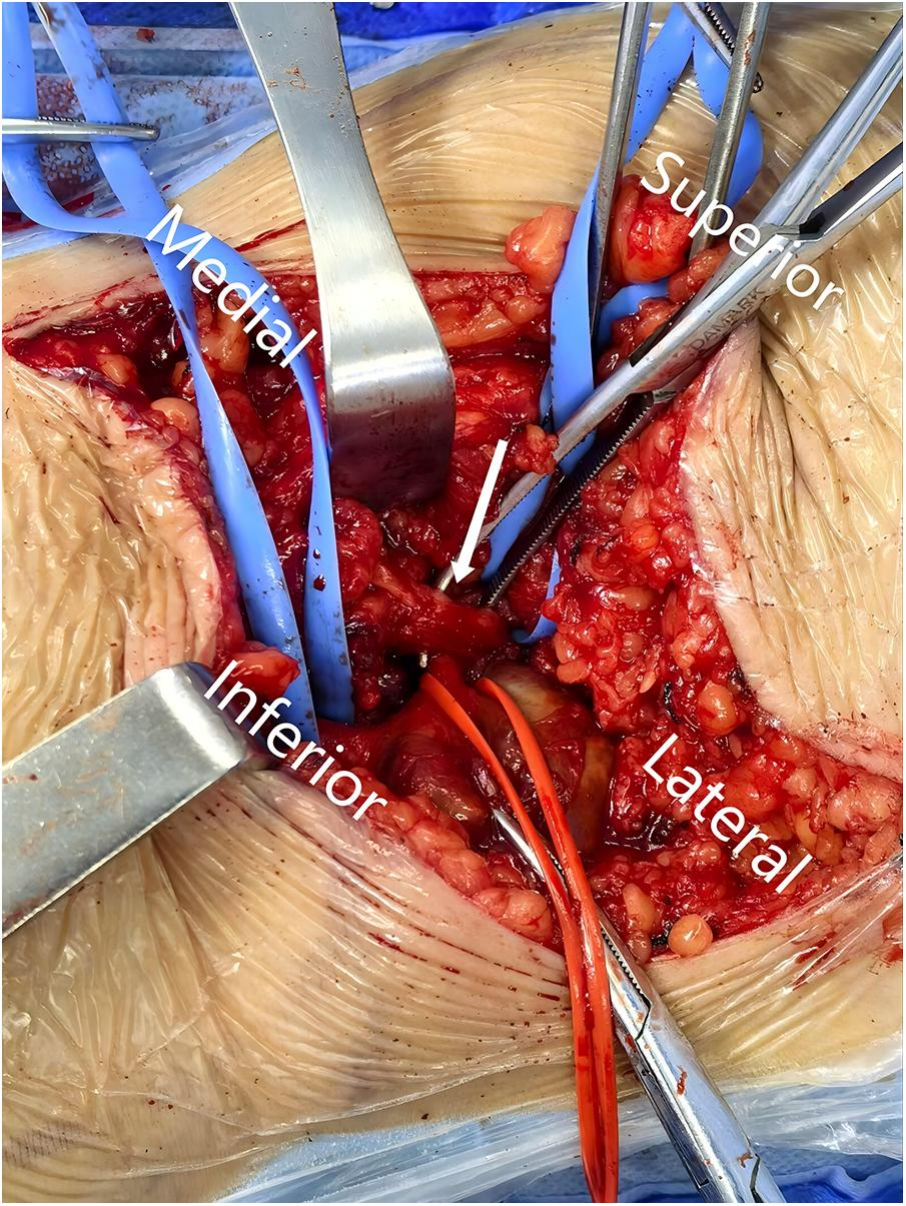
Arrow indicates the medial head of the gastrocnemius muscle compressing the popliteal artery.

The patient underwent resection of a popliteal artery aneurysm and partial resection of the popliteal artery with autologous vein graft replacement under general anesthesia. Intraoperatively, the popliteal artery was found to be eccentrically dilated, compressed by the medial head of the gastrocnemius muscle, resulting in vascular wall deformation and occlusion (Figure 3). The intraoperative diagnosis was popliteal entrapment syndrome. The medial head of the gastrocnemius muscle was partially transected to relieve compression on the popliteal artery, allowing full mobilization of the vessel. The popliteal artery aneurysm was incised, and a large amount of old thrombotic material was removed. The eccentric portion of the aneurysmal wall was resected, while the relatively normal part of the wall was preserved. A segment of the small saphenous vein was harvested, split longitudinally, and trimmed to serve as a patch. A popliteal artery patch angioplasty was then performed. Postoperative pathological diagnosis revealed partially irregular arterial wall tissue, focal fibrinoid material, discontinuity of elastic fibers in the vascular wall, proliferation of smooth muscle and fibrous tissue with hyaline degeneration, scattered hemosiderin deposition, and infiltration of macrophages and lymphocytes.

## 3 Discussion

### 3.1 Overview

Popliteal artery entrapment syndrome (PAES) is a relatively rare disorder in which aberrant anatomical structures around the popliteal fossa compress the popliteal vessels, gradually leading to lower-limb ischemia. Among all PAES patients with imaging documentation, only 9% develop an aneurysm or diffuse arterial dilatation—far less frequent than occlusion (36%) or dynamic stenosis (32%) (1). In young and middle-aged men who report intermittent claudication of the calf or foot, PAES must be suspected. Early manifestations are typically calf tightness or cramping during exercise. As the disease progresses, high-grade popliteal-artery stenosis or occlusion develops, resulting in worsening claudication, rest pain, and, in severe cases, distal limb ischemia and necrosis (2). Occasionally the popliteal vein or tibial nerve is also compressed, leading to chronic venous insufficiency or neurologic deficits, but ischemic symptoms from arterial involvement are by far the most common presentation (3). If dilatation distal to a popliteal-artery stenosis produces a popliteal aneurysm, a pulsatile mass may be felt; conversely, any popliteal aneurysm discovered in a young or middle-aged patient should prompt consideration of PAES. Our 50-year-old patient had an imaging-proven popliteal aneurysm and exercise-induced numbness and pain—typical features of PAES—so the possibility of PAES complicated by PAA must be entertained.

### 3.2 Pathogenesis

Histologically, the popliteal artery resembles large elastic arteries more than a typical muscular peripheral artery; consequently, its aneurysm formation follows a mechanism very similar to that of abdominal aortic aneurysms (4). An imbalance in collagen and elastin metabolism predisposes the artery to dilatation, while inflammation accelerates the degradation of these two proteins and apoptosis of medial smooth-muscle cells impairs wall repair; together these processes culminate in aneurysm formation (5). In addition, mechanical factors are thought to play an important role in aneurysm development: the shear stress produced by hypertension and turbulent flow distal to a relative stenosis can lead to dilatation of the downstream arterial segment (6). Downstream of the entrapment segment, blood that was originally laminar is forced into high-velocity jets and vortices. This alternating “high-low-high” velocity pattern produces markedly increased instantaneous shear stress on the endothelium, damaging the vessel wall and inciting an inflammatory response that promotes aneurysm formation.

In this patient CTA revealed moderate-to-severe stenosis immediately proximal to the popliteal aneurysm, and at operation the artery was shown to be compressed by the medial head of the gastrocnemius; these findings indicate a PAES-induced PAA. Whenever a young, physically active person presents with an isolated popliteal aneurysm or isolated popliteal occlusion and there is no evidence of systemic atherosclerosis, popliteal artery entrapment syndrome should be regarded as the most likely etiology unless proven otherwise (7).

### 3.3 Imaging evaluation

Based on medical history and physical examination, the diagnosis of popliteal artery aneurysm and PAES is generally not difficult, yet it is prone to missed or misdiagnosis. For instance, in this patient, the exacerbation of lower limb pain after exercise and the disappearance of the dorsalis pedis pulse could easily lead to a diagnosis of lower limb atherosclerotic occlusive disease, while overlooking key factors for diagnosing popliteal artery aneurysm or PAES such as the patient's age and the presence of a pulsatile popliteal mass.

In the imaging work-up of popliteal artery aneurysm, duplex ultrasound (DUS) has proved highly sensitive and specific, with reported diagnostic accuracies approaching 100% (8, 9). It should therefore be performed routinely in all patients with suspected popliteal artery aneurysm. In addition to the standard popliteal-aneurysm work-up, this patient underwent electromyography to look for nerve involvement. The study showed a prolonged F-wave latency in the right anterior tibial nerve, suggesting a proximal conduction abnormality. Given the location of the aneurysm, mechanical compression of the common peroneal nerve or one of its branches (e.g., the anterior tibial nerve) cannot be excluded. However, no systematic data exist on the sensitivity or specificity of EMG for detecting nerve compression related to popliteal artery aneurysms; its clinical value awaits further investigation. Because PAES is uncommon it is often overlooked during diagnostic work-up; a definitive pre-operative diagnosis should therefore be built from a combination of tests—including positional stress testing, duplex ultrasound, magnetic resonance imaging (MRI), computed tomography (CT), CT angiography and conventional angiography (10). However, as this case illustrates, multidetector-row CT (MDCT) alone can also provide crucial early diagnostic information for PAES. Magnetic resonance imaging and magnetic resonance angiography (MRI/MRA) clearly depict the soft-tissue structures, allow precise morphologic assessment, and help distinguish functional from anatomic PAES, thereby guiding the choice of surgical approach (11). At present, combined MRI and MRA is regarded as the optimal imaging modality for the diagnosis of PAES (12, 13). Digital subtraction angiography offers limited value in the evaluation of popliteal vascular entrapment syndrome and has now been superseded by non-invasive imaging techniques (14).

### 3.4 Treatment and prognosis

Therapeutic options for popliteal artery aneurysm (PAA) include both open surgery and endovascular repair. However, because the popliteal artery lies across the knee joint, repeated flexion and extension cause covered stents to undergo continual stress; consequently, endovascular treatment is associated with high complication rates and poor durability, so open surgery is generally preferred. In patients with a large PAA and compressive symptoms, a posterior approach is advantageous because it allows complete decompression of the aneurysm sac (8). In this patient the aneurysm measured 4.1 × 3.6 × 3.0 cm, fulfilling the criteria for a giant popliteal artery aneurysm, so a posterior approach was selected. Symptomatic PAES should be treated surgically; endovascular therapy is not appropriate for this disorder. Reports have shown that endografting of a PAES-related PAA without prior decompression leads to early graft failure and may further compromise outflow through thrombosis or distal embolization. Di Marzo, for example, described a PAES patient with arterial occlusion in whom stent-graft placement ultimately failed. Therefore, even though endovascular techniques are now mainstream, the diagnosis of PAES should prompt prompt open decompression and autologous-vein reconstruction of the popliteal artery rather than reliance on endoluminal methods (15). When the popliteal artery is markedly degenerated or occluded, complete replacement with a venous graft is indicated, whereas patients with only “functional compression” should undergo decompression only if they develop clear and typical symptoms (16). At present, no dedicated surgical guideline or standardized operative protocol specifically addresses the combination of PAES and popliteal artery aneurysm (PAA). Nevertheless, clinical practice has established a clear therapeutic principle: patients presenting with both PAES and PAA should be treated primarily by popliteal artery release combined with autologous vein bypass. Multiple series have reported the successful use of small-saphenous-vein bypass grafting for PAES-induced PAA (17). Conventional open procedures usually entail complete excision of the aneurysm or exclusion of the sac combined with bypass. In the present case, however, only the eccentric portion of the wall was resected, leaving part of the native sac *in situ* to preserve local anatomy. When PAES is present this strategy minimizes additional disruption of the popliteal fossa and lowers the risk of post-operative neurovascular complications. Two-month postoperative follow-up: The patient reported no rest pain, coolness, or numbness in the lower limb; walking distance increased from 100 to 200 m preoperatively to 1 km, with only mild fatigue after strenuous activity. Preoperative radiating numbness and pain had completely resolved, objective symptoms were markedly alleviated, confirming the efficacy of the operation and a clear improvement in quality of life.

## 4 Conclusion

This case confirms that popliteal artery entrapment syndrome must be considered in any young or middle-aged patient presenting with an isolated popliteal artery aneurysm. Via a posterior approach we resected only the most diseased portion of the aneurysm wall, preserved the relatively normal segments, and restored a wide lumen with autologous small-saphenous-vein patch angioplasty. This technique promptly relieves arterial compression, minimises dissection within the popliteal fossa, provides durable revascularisation, and obviates the need for complete graft replacement.

The institutional review board of Xuanwu Hospital, Capital Medical University waived ethical approval for this single case report. Written informed consent was obtained from the patient for publication of this report and any accompanying images.

## Data Availability

The original contributions presented in the study are included in the article/Supplementary Material, further inquiries can be directed to the corresponding author.
